# Experiences of patients receiving Home Care and living with polypharmacy: a qualitative interview study

**DOI:** 10.3399/BJGPO.2021.0181

**Published:** 2022-04-20

**Authors:** Stina Mannheimer, Monica Bergqvist, Pia Bastholm-Rahmner, Lars L Gustafsson, Anikó Vég, Katharina Schmidt-Mende

**Affiliations:** 1 Stockholm Region and Division of Family Medicine and Primary Care, Academic Primary Healthcare Centre, Stockholm, Sweden; 2 Division of Nursing, Department of Neurobiology, Care Sciences and Society, Karolinska Institute, Stockholm, Sweden; 3 Karolinska Institute, Division of Clinical Pharmacology, Department of Laboratory Medicine, Karolinska University Hospital, Stockholm, Sweden; 4 Pedagogue, Region Stockholm, Health Care Administration, Stockholm, Sweden; 5 Karolinska Institute, Department of Neurobiology, Care Sciences and Society, Karolinska University Hospital Huddinge, Sweden

**Keywords:** general practice, health services for the aged, polypharmacy, multimorbidity, frailty, psychosocial functioning, primary healthcare

## Abstract

**Background:**

In Sweden, patients receiving Home Care (HC) are older people with frailty and multimorbidity, and are often treated with many medicines. Their perspectives on polypharmacy have been sparsely explored.

**Aim:**

To investigate HC patients’ experiences and perceptions regarding polypharmacy.

**Design & setting:**

Semi-structured interviews with 17 patients with HC in Stockholm, Sweden.

**Method:**

The interview questions were open and aimed to encourage participants to speak freely about their personal experiences of living with polypharmacy. Data were analysed using an inductive thematic analysis.

**Results:**

The participants’ median age was 83.5 years (range 74–97 years) and the median number of prescribed medicines was 11 (range 5–30). The following two themes were identified: (1) experiences from daily life with polypharmacy; and (2) dependency on the relationship to healthcare professionals. The first theme contains the main finding, which was the diversity in how older people experienced polypharmacy and how they coped with polypharmacy in everyday life. While some were satisfied despite having multiple medicines, others experienced such psychological unease owing to polypharmacy that it led to reduced quality of life. The second theme reflects the importance of the relationship between the older person and healthcare professionals for medicine-related ideas and attitudes.

**Conclusion:**

The individual variation in experiences regarding polypharmacy points to the value of interprofessional teamwork with the patient as an active partner. Therefore, healthcare professionals need to adapt a more person-centred approach where the patient’s perspectives are respected and considered in medicine-related decisionmaking.

## How this fits in

A limited number of qualitative studies have been conducted on how older people perceive polypharmacy, and few have had participants with as much frailty and multimorbidity as HC patients. Given an ageing population and widespread use of multiple medicines, knowledge on how this group experience polypharmacy is crucial to improve conditions for person-centred care and thus enhance health outcomes.

## Introduction

Prescriptions of medicines to Swedish people aged >75 years have increased by approximately 60% since the beginning of the 1990s,^1^ and about every other person in this group uses at least five medicines.^2^ Likewise, the occurrence of polypharmacy is widespread among older adults throughout high income countries.^3,4^ While polypharmacy may be appropriate and improve health, the risk of a range of adverse health outcomes is increased with the number of medicines used, particularly for older patients with multimorbidity.^3^ Older people often trust GPs’ expertise regarding medicines.^5,6^ Still, complex medicine regimens and uncertainties in decisionmaking are perceived as challenging by many older patients as well as GPs.^7^ Several studies reveal dislike or even reluctance regarding intake of medicines, and a preference to take as few medicines as possible.^8^ Older people may refrain from sharing information about their own initiatives to discontinue medicines with their GP,^9^ which can be hazardous. Previous research has highlighted the need for improved communication between primary healthcare professionals and older patients by making therapeutic relations more horizontal,^10^ which implies that healthcare professionals must support older people to manage drug therapy based on their own conditions. HC patients are a group of particularly frail older people (see Box 1), in whom polypharmacy is common. The authors are not aware of any previous study exploring HC patients' experiences of polypharmacy. However, this matter is important in order to improve patient–healthcare practitioner relations and quality of care.

Box 1 **Home care in Swedish primary health care**

Home care systems differ between countries in Europe.^33^ The Swedish home care (HC) system, operated by nurses and physicians from local primary healthcare centres,^34^ is intended for older adults with frailty and multimorbidity, who have considerable need for primary health care and experience difficulties visiting GP practices. These patients commonly have both medical and functional problems.^35^ In Sweden, a patient will be registered as an HC patient if a district nurse’s assistance is required more than twice per month. Regular visits from a district nurse enable older people with multimorbidity, with reduced functional capacity and/or cognitive impariment, to receive care in a familiar context and maintain their independence. This close contact may also diminish the need for visits by the GP.^36^ In 2019, 19 000 people in Region Stockholm received HC. The typical Swedish HC patient is aged 82 years, has four chronic diseases, and is treated with 13 different medicines. These patients are often granted support from the municipal HC services, which offer daily assistance to enable older people to live at home. When functional decline worsens, HC patients may need nursing home care.

## Method

### Study design

This study explored how HC patients experience their drug therapy, with focus on living with polypharmacy. In accordance with the aim, a qualitative study design was chosen with inductive thematic analysis^11^ of semi-structured interviews.^12^ The consolidated criteria for reporting qualitative research (COREQ) was followed.^13^

### Selection of participants

The research team contacted primary healthcare practices in suburban areas with different socioeconomic status in the Stockholm Region. Through local HC nurses, contact was made with HC patients. The patients approached had at least five prescribed medicines and were assumed to be able to participate in a long interview, which would require a sufficient verbal and cognitive ability. The patients had no dementia diagnosis or advanced stage of terminal illness. Of 19 responders, 18 agreed to participate and were scheduled for an interview. Owing to hospitalisation of one patient, 17 interviews were conducted, of which six were with men (median age 84.5 years) and 11 with women (median age 82 years). For participant characteristics, see Supplementary Box S1.

### Data collection

The individual interviews were conducted in the patients’ private homes in 2018 and 2019. The interviews were conducted by four people: SM (resident GP), KSM (GP), PBR (behavioural scientist), and JS (resident GP). None of the interviewers had any kind of relationship with the participants and had not met them before the interviews.

To understand different aspects about how the patients experience their drug therapy, the interviews started with broad open-ended questions (see Supplementary Box S2 for the interview guide). Based on the answers, follow-up questions were used to clarify and deepen the participant’s answer. By asking questions such as, 'What do you mean?' or 'Could you give an example?', information reflecting the participant’s experiences was gained on a deeper level. The interviews lasted between 35 and 75 minutes, were audio-recorded and transcribed verbatim. During the last interviews participants’ comments tended to be reiterative of data already collected, thus saturation was assumed.^14^

### Data analysis

From the empirical material, an inductive thematic analysis was carried out.^11^ In this approach, the categories and themes identified are strongly linked to the data without trying to fit them into a pre-existing theoretical frame.

The phases of the analysis were as follows:

1. The transcripts were read several times by two researchers to acquire a good sense of the whole material.
2. Sections of text and keywords in the transcripts, with focus on the aim of the study, were marked and coded. Marked sections with related topics were grouped into categories. Categories were then compared for emerging patterns of similarities and differences, which was carried out separately by two researchers and then discussed.
3. The next step was to find related patterns within each category. Certain categories were considered to have a common origin, were related, or both. When opinions differed between research members, the authors returned to the transcripts and discussed them until an agreement was reached (negotiated consensus). This was a way to reach consensus of the findings and trustworthiness of the data analysis.
4. The categories were then merged into two themes. These themes are interconnected and cannot be seen as isolated because they are closely related to each other.
5. Quotes were selected to illustrate each category.

The data analysis was mainly performed by SM and MB (registered nurse). KSM, PBR, and AV (pedagogue) took part in the analysis process of finding related patterns between the categories and themes through a reciprocal reading between transcribed text and the categories and themes. This is a method to increase the trustworthiness in the analysis process.^15^ All authors then acted as co-readers to finally discuss and refine the findings in relation to the research questions. The main aim of this process was to compare the results to determine whether any categories or themes had been overlooked.

### Ethical considerations

Oral and written informed consent was obtained from every participating patient before the interview, as well as from the head physician at the patient’s primary healthcare centre. Before each interview, the patients were again informed of the aim of the study and that participation was voluntary and could be discontinued at any time. After transcription, the identities of the participants were coded to guarantee confidentiality, and the audio files were deleted.

## Results

**Figure 1.**
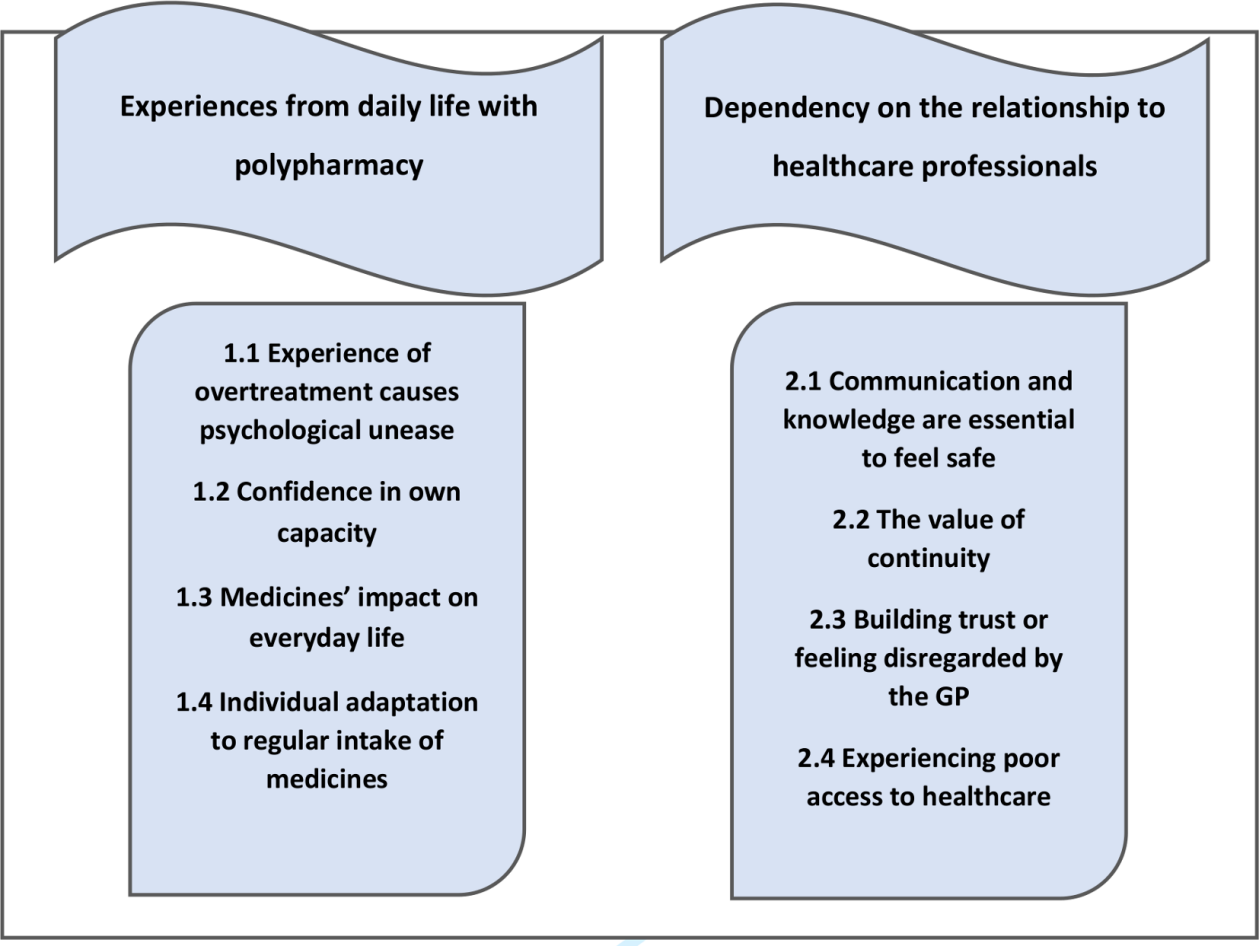
Patients with HC’s experience of polypharmacy: themes 1 and 2 (banners), with categories below

The analysis of 17 interviews with HC patients resulted in two themes with underlying categories (see Figure 1).

### Theme 1: Experiences from daily life with polypharmacy

Participants demonstrated different strategies to cope with polypharmacy in everyday life. Some were satisfied with their complete medicine therapy, whereas others found it hard to accept the need for medicines. The participant's trust in their own capacity to handle their medicines varied, and several challenges were revealed. Some experienced having too many pills, which could cause concern and anxiety.

#### 1.1 Experience of overtreatment causes psychological unease

Many participants experienced having too many pills. They were uncertain about how the drug therapy affected their body and found it hard to keep track of medicines from day to day. For some, this feeling was overwhelming and resulted in concern and resignation. Some felt forced to consume medicines, and felt that their opinion did not matter. One woman described feeling anxious and powerless, as she felt forced to take pills prepared for her, and another was afraid they would develop depression from all medicines and considered quitting altogether:

*'One can’t refuse, can you? If they have decided that it would be good if you tried this medicine too, then you cannot say, “No, I don’t want more.”'* (Informant 12; man, 74 years, 30 medicines)

Dissatisfaction with polypharmacy led to increased need for autonomy regarding medicines. One participant reduced the number of pills by discontinuing one medicine at a time without informing their doctor. If there was no symptom relapse, they might not resume the treatment:

*'I’m thinking does one really need these pills … I just feel “no”. No, I won’t take this pill today, I will see tomorrow. That’s actually how I do it! It’s trial and error for me. Not just one day, it has to be a couple of days because one day is nothing. So I try it out and sometimes I feel better and sometimes worse.'* (Informant 4; woman, 74 years, 13 medicines)

#### 1.2 Confidence in own capacity

Some participants described how they consciously deviated from the doctor’s recommendations regarding medicine dosing, to better fit their daily activities. Several participants trusted their own capacity to handle their medicines and could differentiate between crucial pills and those that could be omitted occasionally:

*'I’m very careful, I have two tablets for the heart, or for the breathing and that, or heart failure or what’s it called. I’m very careful with those, but the others, if I were to forget some of them one day then I know that that one, the gout, I can do without and that other one I can do without.'* (Informant 8; woman, 86 years, 14 medicines)

Several participants were aware that discontinuation of medicines may cause disease relapse, which increased their motivation to pursue their drug therapy:

*'I have had lithium for many, many years. I was depressed long ago. One doctor terminated that treatment, and it was a disaster. So I will probably take it for the rest of my life, until I die so to speak.'* (Informant 14; man, 90 years, seven medicines)

#### 1.3 Medicines’ impact on everyday life

Participants who experienced allergic symptoms, diarrhoea, loss of hair, faecal and urinary incontinence, nausea, dizziness, or fatigue sometimes suspected side effects. A woman who suffered from diarrhoea as a presumed side effect described that her symptoms were not taken seriously by healthcare professionals. Moreover, she experienced social isolation as she did not dare to leave her house:

*'I have told them, I mean I do not dare to go out, because several times it just … I had to throw myself in the car and drive home because of the pain. It is like a cramp in the stomach, I felt it, but it just poured down. Shoes and clothes ... I was at my son’s place for dinner.'* (Informant 7; woman, 80 years, 10 medicines)

#### 1.4 Individual adaptation to regular intake of medicines

Some participants were sceptical towards medicines whereas others found them easy to accept, but in the end, many were thankful for medicines. Once integrated in everyday life, initial doubt or mistrust decreased:

*'Without the medicines I wouldn’t be here. I believe in my medicines, that it helps and makes me stay the way I am and even become healthier.'* (Informant 11; woman, 85 years, nine medicines)

Still, participants stated that living with polypharmacy was an everyday challenge in practical terms. Challenges could include remembering when to take medicines, opening the packages, dispensing doses, and administering them correctly. Several participants used a multi-dose medicine dispenser, a 'pill organiser', which they filled themselves or got filled regularly by the HC nurse. Others used the medicine dispensing system 'Apodos', a dosing aid with disposable plastic bags containing all pills for 1 or 2 weeks’ use. Still, several participants regarded it as problematic that they forgot to take their medicines, which commonly occurred with pills that should be taken between meals:

*'The worst thing is that if I should take four, one is at midday when I don’t eat. And that’s the one I forget sometimes.'* (Informant 14; man, 90 years, seven medicines)

Pill organisers or Apodos were perceived as useful tools. However, the practical handling of plastic bags was challenging, thus working against the correct intake of medicines:

*'And now with these bags, I cut them open with my injured hand and empty them. Then I sit here and fill my pill organiser. When that is done, I count. Did I fill in all six? Sometimes, I might have dropped one. This one is washed with chlorine* [pointing out a flyswatter]*, I grab the swat and pick it up from the floor.'* (Informant 9; woman, 77 years, six medicines)

### Theme 2: Dependency on the relationship to healthcare professionals

The participants conveyed a range of experiences from their relations to professionals within the healthcare system. Commitment from healthcare professionals and sincere communication were identified as crucial for the transfer and receipt of knowledge. Some expressed trust and satisfaction, while others had negative experiences of contacts with healthcare professionals, which caused frustration and feelings of powerlessness.

#### 2.1 Communication and knowledge are essential to feel safe

Many participants described good communication with their GP. They felt listened to and received useful information. However, in cases of poor communication and uncertainty over what had been said, participants found it difficult to participate actively in their own care and treatment. One participant was uncertain of when to take their medicine for angina since she was unsure of the symptoms of angina:

*'My GP has given me something else that she wants me to take when I have angina, is that what it’s called? But frankly I don’t know when I have angina, still I take it occasionally.*' (Informant 8; woman, 86 years, 14 medicines)

There were also uncertainties regarding the reasons for treatment with some medicines. Indication for each medicine is documented in the patient’s medicine list, but still, this information is seldom phrased in a way that the patient understands. One participant exclaimed:

*'… and hypokalemic what is that? Hell knows … what that means.'* (Informant 7; woman, 80 years, 10 medicines)

Another participant believed that medicines gave rise to further diseases and was afraid of a vicious circle:

*'Yes, they could spend more time looking into the fact that one takes too many medicines. I mean, it’s not good for the body either. And there will be another disease added on when you get that medicine. Another disease will come. And when it goes on, there will be no improvement. It just gives rise to yet another.'* (Informant 4; woman, 74 years, 13 medicines)

#### 2.2 The value of continuity

Several participants were satisfied with the continuity of care and expressed genuine trust in their GPs and the HC nurses, who some of them had known for years. They reported that the quality of the interaction with healthcare professionals in many ways was dependent on the personal contact and if you had had the chance to get acquainted. Moreover, the attitude towards certain medicines was influenced by the relationship to the prescribing physician:

*'I would never do that* [stop taking medicines without telling the doctor]*. No. If you only do what he tells you to, everything will be fine.'* (Informant 3; woman, 81 years, 11 medicines)

Some participants who had received medical care and prescriptions from several physicians were concerned and uncertain, especially when physicians in both primary and secondary care had made alterations to their prescriptions. One participant felt as if they had one specialist for each organ, but no one who had the overall responsibility for their medicines:

*'I had medicines for something that I didn’t need and since many doctors were involved it became confusing, that’s how I experienced it.'* (Informant 6; woman, 90 years, 15 medicines)

In several cases, participants expressed trust in the HC nurses, who coordinated care and with whom they could discuss medicine-related issues. Some experienced better continuity with nurses than with physicians, and the cordial relationship with the HC nurse was highly esteemed:

*'I have the nurse who is my… my friend. She likes to come herself to give the medicines. Every 14* ^*th*^ *day they do it for me, I don’t know how to do it since I cannot see.'* (Informant 8; woman, 86 years, 14 medicines)

#### 2.3 Building trust or feeling disregarded by the GP

Several participants requested increased commitment from their GP in terms of active listening. One participant described an appointment with a GP as follows:

*'When I was at my appointment, the doctor stared into that computer screen while talking to me.* […] *I have heard so many who get irritated with the physicians, they just sit and glow at the screen.'* (Informant 6; woman, 90 years, 15 medicines)

Another participant felt overridden when the GP had not paid attention to what they regarded as the main problem:

*'She did not care what I had come there for, my knee, but it was all about the diabetes. I was a little mad at her and said I wanted to change doctor, because she did not really listen.'* (Informant 3; woman, 81 years, 11 medicines)

Participants who experienced absence of effect or side effects of their drug therapy expressed that this was dismissed by the GP who attributed the symptoms to the process of natural ageing:

*'The doctor knows medicine, naturally, but the patient is the one with the best knowledge of his body. It was like they believed in the test results, the blood samples, more than in me, my body.'* (Informant 2; man, 88 years, 13 medicines)

#### 2.4 Experiencing poor access to health care

Some participants experienced that over the years, it had become more and more difficult to get in touch with their GP. This raised feelings of mistrust and ambivalence. When they finally got in contact and had obtained an appointment, consultation time was too short for questions or discussion:

*'Earlier on you got invitations to appointments one or twice a year. Now you must demand to be let in. And I must call and ask for my prescriptions when they have run out. You ought to have knowledge about everything and thank God I still have a clear mind.'* (Informant 2; man, 88 years, 13 medicines)

One participant expressed empathy with the GPs because of the heavy workload that hampers continuity:

*'It can’t be easy to be a doctor nowadays in primary health care. They are obliged to see an awful lot of patients; they never have time to get emotionally involved. I wouldn’t want to be a doctor and work under those circumstances, as they do here. I understand them though, they don’t have time to sit with me and go through pills.'* (Informant 6; woman, 90 years, 15 medicines)

## Discussion

### Summary

The aim of this study was to gain an understanding of how patients in HC experience polypharmacy in daily life. The first main finding is the considerable variation in experiences from living with polypharmacy. For some patients, multiple medicines had become part of their daily routine and was not perceived as problematic, whereas for others, it was associated with great unease. The individual differences point to the second main finding, which is the importance of sincere communication in the patient–GP relationship in order for these patients to feel safe regarding drug therapy. The third main finding is that participants in the study experienced improved continuity with the healthcare unit through regular contact with HC nurses, who were regarded as a significant support and had a coordinating role working as a bridge to the GP. Several participants experienced a more informal relationship with the HC nurse than with the GP, which could be an underutilised opportunity for discussions about medicine-related experiences and points to the value of interprofessional teamwork.

### Strengths and limitations

To the best of the authors' knowledge, this is the first study to qualitatively explore experiences from polypharmacy in daily life in a population experiencing HC, as well as how these patients perceive contacts with healthcare professionals. It is a strength that interviews were carried out older people with multimorbidity and fraility in their own homes, as the authors believe that this context allowed the participants to feel secure and to speak freely. The sex distribution with 11 women and six men corresponds to the distribution in the whole HC population in Stockholm and even in the Nordic countries,^16^ which enhances transferability of findings. However, transferability is reduced by the fact that no patients with dementia were included, as this diagnosis is common in the HC population. Therefore, the research team plans to complement this research with a study that includes patients with dementia through interviews with relatives. The fact that interviews were conducted by four interviewers could be a limitation; however, this was handled through a shared interview guide, as well as close cooperation within the multiprofessional team through the analysis. It is believed that the team members’ different experiences and competences from the medical, psychological, and sociological fields have contributed constructively to reflexivity and the interpretation of data. The trustworthiness of the findings is strengthened by use of multiple researchers engaged in each of the analytical phases.

### Comparison with existing literature

A considerable variation in experiences of polypharmacy was identified. This is in line with findings in a systematic review of qualitative studies from 2020,^17^ highlighting the importance for healthcare professionals to actively solicit individual patient's perspectives on drug therapy to facilitate shared decisionmaking. Participants in the current study presented different reasons for deviating from recommendations regarding medicine dosing. Regardless of the cause, these patients need to be offered support from the GP to make informed choices about their medicines. This is reflected in findings from an English study, which suggested that healthcare professionals should guide patients to ‘get to know’ their medicines and how to administer them to improve health outcomes safely.^18^ However, owing to negative experiences with physicians regarding the ability to discuss concerns about medicines, previous findings show that patients tend to seek alternative sources of information that may, in the end, entail medical risks.^19^ In this context, the present study's finding that patients may not dare to question physicians about medicines is of great concern and must be addressed.

Findings regarding communication and continuity of care are in line with previous research in the older population. Several studies from Sweden and other European countries have identified the patient–GP relationship as central for multimorbid older patients’ views on medicines; still, these studies did not include HC patients as a specific group.^20,21^ Most participants in the study trusted their GPs, but nevertheless some felt unable to communicate concerns regarding medicines. Thus, more than trust seems to be required for HC patients to participate in their own treatment and to feel safe in the patient–GP relationship. A study from Denmark suggested that unequivocal trust in the GP may prevent patients from asking questions and discussing medical decisions, and that power imbalances and lack of continuity of care may be more important to address.^22^ A relationship characterised by trust but also healthy questioning and open communication, seems to enhance older people's participation in their own health care.^23^

Regarding interprofessional teamwork, nurse-led interventions have shown to improve medicine adherence among home-dwelling older adults newly discharged from hospital care.^24^ Several studies point to the value of nurses for pharmacovigilance, particularly for identifying adverse drug reactions.^25,26^ However, to achieve an improved identification of potential obstacles and risks, it is suggested that nurses' specific knowledge on the topic should be advanced through in-service training and education,^27^ and that awareness of this matter should be improved among healthcare students.^28^

### Implications for practice

The results have shown that older people were not always encouraged or involved in shared decisionmaking by the GP, even if they showed interest in medicine-related issues. Still, a large proportion of older people wished to be actively involved in their drug therapy, which has been described as a potential method to enhance patient safety.^29^ Shared decisionmaking has also been associated with improved health outcomes.^30^ Person-centred care has emerged as an area of growing interest in the past decade,^31^ but it remains to be clarified how a person-centred approach should be implemented for HC patients with frailty.^32^
